# Electromagnetic Modulation Signal Classification Using Dual-Modal Feature Fusion CNN

**DOI:** 10.3390/e24050700

**Published:** 2022-05-15

**Authors:** Jiansheng Bai, Jinjie Yao, Juncheng Qi, Liming Wang

**Affiliations:** 1 State Key Lab for Electronic Testing Technology, North University of China, Taiyuan 030051, China; b200504@st.nuc.edu.cn (J.B.); yyyjinjie@163.com (J.Y.); qijuncheng@nuc.edu.cn (J.Q.); 2 School of Information and Communication Engineering, North University of China, Taiyuan 030051, China

**Keywords:** automatic modulation classification, feature fusion, gram angular field, deep learning, convolutional neural network

## Abstract

AMC (automatic modulation classification) plays a vital role in spectrum monitoring and electromagnetic abnormal signal detection. Up to now, few studies have focused on the complementarity between features of different modalities and the importance of the feature fusion mechanism in the AMC method. This paper proposes a dual-modal feature fusion convolutional neural network (DMFF-CNN) for AMC to use the complementarity between different modal features fully. DMFF-CNN uses the gram angular field (GAF) image coding and intelligence quotient (IQ) data combined with CNN. Firstly, the original signal is converted into images by GAF, and the GAF images are used as the input of ResNet50. Secondly, it is converted into IQ data and as the complex value network (CV-CNN) input to extract features. Furthermore, a dual-modal feature fusion mechanism (DMFF) is proposed to fuse the dual-modal features extracted by GAF-ResNet50 and CV-CNN. The fusion feature is used as the input of DMFF-CNN for model training to achieve AMC of multi-type signals. In the evaluation stage, the advantages of the DMFF mechanism proposed in this paper and the accuracy improvement compared with other feature fusion algorithms are discussed. The experiment shows that our method performs better than others, including some state-of-the-art methods, and has superior robustness at a low signal-to-noise ratio (SNR), and the average classification accuracy of the dataset signals reaches 92.1%. The DMFF-CNN proposed in this paper provides a new path for the AMC field.

## 1. Introduction

Automatic modulation classification (AMC) is automatically identified the modulation scheme of the received electromagnetic signal [1,2], which plays an essential role in spectrum monitoring, malicious electromagnetic signal identification, electromagnetic interference identification and other fields. With the development of wireless communication, the modulation types of electromagnetic signals vary, and the wireless environment is also worse, making AMC more difficult. Therefore, it is essential to explore more effective AMC methods. In recent years, the advantages of AMC methods based on deep learning in feature extraction and classification accuracy have received extensive attention, but they also face the problem of how to characterize the original electromagnetic modulation signals properly. Specifically, before inputting the original signal into the deep neural network, it is necessary to combine the signal characteristics for preprocessing and design a superior neural network structure to improve AMC’s performance, which is a topic worthy of study.

AMC methods of electromagnetic signals can be divided into two types: (1) likelihood-based (LB); (2) feature-based (FB) [3]. LB optimizes the classification accuracy according to the likelihood function [4] (such as mixed likelihood ratio, average likelihood ratio and generalized likelihood ratio) of different modulation signals. However, the complexity and classification accuracy of the AMC algorithm are negative impacts of the number of signal types. The computational complexity of FB is low, and a better classification effect can be achieved by reasonably designing feature extraction methods. The FB classification algorithm generally includes feature extraction and a classification network. Many researchers have studied various features for extraction, including high-order cumulant (HOC) [5], cyclic spectrum [6], approximate entropy [7], Kullback–Leibler divergence (KLD) [8], etc. Classification networks include the artificial neural network (ANN) [9], decision tree, support vector machine (SVM) [10] and k-nearest neighbor (KNN) [11]. In the case of multi-type modulation signal recognition and classification, the above methods have the problems of manual feature extraction and time-consuming operation. At the same time, they have poor adaptability in an environment of low SNR.

To solve the above AMC problems, we can take advantage of the powerful feature extraction ability and classification accuracy of deep learning (DL) [12]. The deep learning network framework plays an important role in signal recognition, and many researches have focused on improving the network framework. For example, combining multiple CNN structures, LSTM and CNN network structures through automatically hyperparameter tuning [13,14,15,16] to improve network performance, etc., has promoted signal recognition work. In the modulation classification method based on DL, to fully use the advantages of CNN’s classification and recognition ability, it has become a research hotspot to preprocess and characterize the received signal in an appropriate form before inputting the signal into the CNN [17]. The original electromagnetic modulation signal can be represented by a signal feature [18,19,20,21,22], image [23,24,25,26] and sequence [27,28,29], but this single-parameter conversion method ignores the complementarity between features and has certain limitations. In response to this problem, some researchers began to combine multiple features for AMC, including multi-type graph feature fusion [30,31,32] and multi-type sequence feature fusion [33,34,35], such as cyclic spectra image and constellation diagrams [30,31], time–frequency diagrams and instantaneous autocorrelation images [32], SPWVD and BJD images [33], high-order cumulant and IQ sequences [34], DOST sequences and IQ sequences [36], etc.

Although the above methods fuse multiple features, there is a common limitation: they do not consider the complementarity between different modal features and integrate them with an appropriate fusion mechanism. The difference and multi-dimensionality between different modalities, such as image and time series data, will further improve the accuracy of AMC. At the same time, most of the existing DL framework-based AMC methods mentioned above try to characterize the original modulated signal, but rarely consider the relationship between the signal features and network structure. Image representation methods combined with CNN have achieved a good recognition effect, but for sequence data, especially IQ sequences, it does not make full use of real and imaginary part information, resulting in the inability to extract the characteristics of the modulation signal fully. Obviously, it is better to extract the characteristics of IQ data in the complex value domain.

### Contributions

To further improve the accuracy of AMC, a modulation classification method based on dual-mode feature fusion CNN is proposed, and GAF [37] theory is introduced into the field of AMC for the first time. It can be summarized that the innovative elements of this study are as follows:

In this paper, the complementarity of different modal features is fully considered. The GAF theory is introduced into the AMC field. The one-dimensional signal is encoded into a two-dimensional image, and the ResNet50 network structure is adjusted to extract the features of the GAF image. At the same time, using the more vital representation ability of complex data than real data, the original signal is transformed into IQ data, and CV-CNN is constructed to extract amplitude and phase features.

A dual-modal feature fusion mechanism (DMFF) is proposed to fuse the features extracted by GAF-ResNet50 and CV-CNN. Furthermore, the DMFF-CNN classification model is trained by combining the fusion features. In the training process, the penalty term is added between the dual-modal feature tags to reduce the network complexity and improve the classification accuracy of DMFF-CNN.

In the experimental part, eight types of electromagnetic modulation signal samples are generated according to different parameters, such as chip rate (CR), carrier frequency, modulation frequency and phase difference, so as to improve the generalization of the existing dataset.

Compared with other advanced methods, the DMFF-CNN model proposed in this paper has achieved excellent and stable results in signal classification experiments. At the same time, it has good robustness in a −10 dB low SNR environment, and the classification accuracy reaches 92.1%.

## 2. Materials and Methods

### 2.1. Overview of the Proposed Method

**Figure 1:**
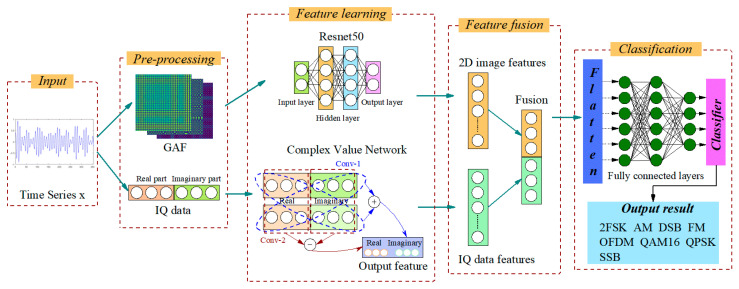
Block diagram of the proposed scheme.

The structure of the DMFF-CNN proposed in this paper is shown in Figure 1, and it is divided into four parts: (1) signal preprocessing; (2) feature extraction; (3) dual-feature fusion; (4) signal classification. In the first part, GAF image coding is used to reconstruct and upgrade the data of the original signal, and convert the one-dimensional sequence into a two-dimensional image, which is used as the input of ResNet50. At the same time, the signal is down-converted to the intermediate frequency band, down-sampled, and then demodulated to obtain IQ data, which are input to CV-CNN. The second part extracts the GAF image and IQ data features by ResNet50 and CV-CNN, respectively. In the third part, the DMFF-CNN is constructed, and the DMFF mechanism is used to fuse different modal features. In the fourth part, the DMFF-CNN classification model is trained and tested by fusion features, and the classification results of electromagnetic modulation signals are obtained.

### 2.2. Dataset Preprocessing

#### 2.2.1. Two-Dimensionalization of the Time Series Signal Based on GAF

GAF is a method of transforming time series into images through time coding [37,38]. The process of converting time series into images is divided into three steps: Firstly, normalize the input time series data to [−1, 1]. Secondly, convert the normalized time series data from the Cartesian coordinate system to the polar coordinate system, and retain the time information of the original input signal. Finally, compare the time correlation of each time point in polar coordinate through triangular cosine function, so as to obtain a matrix with *n* × *n* dimension, where *n* is the number of sampling points in time series data.

Suppose a time series signal expression as $X=\left\{x_{1},x_{2},\cdots ,x_{n}\right\}$, which is a sequence composed of n time points and corresponding actual observations *x*. In order to reduce the bias of the inner product to the maximum value, the Min-Max scaler is used to scale the time series to within [–1, 1]. The formula for the Min-Max scaler is as follows

(1) $$\tilde{X_{0}^{i}}=\frac{x_{i}-min\left(X\right)}{max\left(X\right)-min\left(X\right)},$$

Then, the value of the scaled sequence *X* is mapped to an angle $\varphi_{i}$, time ‘*t*’ is mapped to radius ‘*r*’. The value of the time series and its corresponding timestamp is represented by $\varphi_{i}$ and *r*, so the scaled time series *X* is redefined in the polar coordinate system

(2) $$\{\begin{array}{c} \varphi_{i}=arccos\left(\tilde{x_{i}}\right),-1\leq \tilde{x_{i}}\leq 1,\tilde{x_{i}}\in \tilde{X}\\ r=\frac{t_{i}}{N},t_{i}\in N \end{array},$$

In (2), $t_{i}$ is the time stamp, and the interval [0, 1] is divided into *N* equal parts to regularize the span of the polar coordinate system. The coding of (2) has two essential properties. Firstly, (2) is bijective because cos(φ) is monotonically decreasing when φ∈[0,π]. Therefore, it is unique in the polar coordinate system, and its inverse mapping is also unique. Secondly, unlike Cartesian coordinates, polar coordinates maintain an absolute temporal relationship.

After mapping the one-dimensional signal to the polar coordinate system, the correlation between adjacent connecting points (i,j) is expressed as (3)

(3) $$i\;⨁\;j=cos\left(\varphi_{i}+\varphi_{j}\right),$$

where $\varphi_{i}$ and $\varphi_{j}$ represent the corresponding angles of vectors *i* and *j* in the polar coordinate system, respectively. The angular perspective is used to identify the correlation of each time point in different intervals, so as to obtain the Gram matrix called the sum of gram angles, as shown in (4)

(4) $$\begin{array}{l} G=\left(\begin{array}{cccc} \cos\left(\varphi_{1}+\varphi_{1}\right) & \cos\left(\varphi_{1}+\varphi_{2}\right) & \cdots & \cos\left(\varphi_{1}+\varphi_{n}\right)\\ \cos\left(\varphi_{2}+\varphi_{1}\right) & \cos\left(\varphi_{2}+\varphi_{2}\right) & \cdots & \cos\left(\varphi_{2}+\varphi_{n}\right)\\ \vdots & \vdots & \ddots & \vdots \\ \cos\left(\varphi_{n}+\varphi_{1}\right) & \cos\left(\varphi_{n}+\varphi_{2}\right) & \cdots & \cos\left(\varphi_{n}+\varphi_{n}\right) \end{array}\right)=,\\ {\tilde{X}}^{\prime }\cdot \tilde{X}-{\sqrt{I-{\tilde{X}}^{2}}}^{\prime }\cdot \sqrt{I-{\tilde{X}}^{2}} \end{array}$$

In (4), *I* is the unit row vector [1,1,⋯1]. After mapping time series to the polar coordinate system, the time series of each step is regarded as 1-D metric space. Because GAF is more sparse, the inner product is redefined in the Cartesian coordinate system $x,y=x\cdot y-\sqrt{1-x^{2}}\cdot \sqrt{1-y^{2}}$. Compared to traditional inner products, newly defined inner products add a penalty term, which can separate the desired output from Gaussian noise. *G* is a Gram matrix, expressed as (5)

(5) $$G=\left(\begin{array}{cccc} \langle {\tilde{x}}_{1},{\tilde{x}}_{1}\rangle & \langle {\tilde{x}}_{1},{\tilde{x}}_{2}\rangle & \cdots & \langle {\tilde{x}}_{1},{\tilde{x}}_{n}\rangle \\ \langle {\tilde{x}}_{2},{\tilde{x}}_{1}\rangle & \langle {\tilde{x}}_{2},{\tilde{x}}_{2}\rangle & \cdots & \langle {\tilde{x}}_{2},{\tilde{x}}_{n}\rangle \\ \vdots & \vdots & \ddots & \vdots \\ \langle {\tilde{x}}_{n},{\tilde{x}}_{1}\rangle & \langle {\tilde{x}}_{n},{\tilde{x}}_{2}\rangle & \cdots & \langle {\tilde{x}}_{n},{\tilde{x}}_{n}\rangle \end{array}\right),$$

GAF contain temporal correlations because $G_{\left(i,j||i-j=k\right)}$ indicates that temporal correlations are superimposed through the direction of time interval *k* and explained by relative correlations. When *k* = 0, the main diagonal $G_{i,i}$ consists of the original values of the scaled time series. The time series of the original signal is approximately reconstructed by using the $G_{i,i}$ and the high-level characteristics extracted by deep learning.

**Figure 2:**
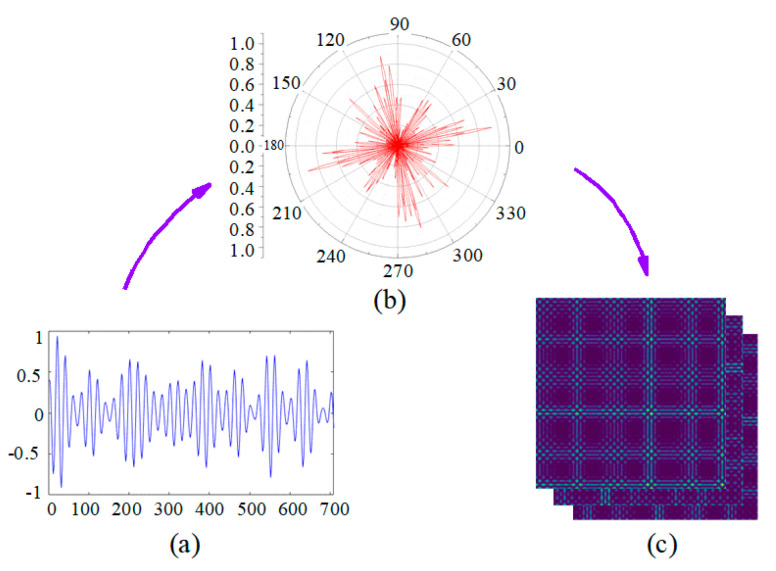
Two-dimensionalization of signal-based GAF. (**a**) Time Series *x*; (**b**) Polar Coordinate; (**c**) Gramian Angular Field.

The GAF algorithm is further illustrated in Figure 2. The original signal is converted into a polar coordinates diagram through (2) as shown in Figure 2b, and the GAF images are transformed using (5); the result is shown in Figure 2c. It can be found that after the 2-D of the signal, the features in the time domain waveform are fully represented in the GAF images, and the electromagnetic modulation signal is fully mapped through different characteristics such as color, point and line at the corresponding position [38].

#### 2.2.2. Conversion of Time Series Signal into IQ Data

Compared with real values, complex values have more robust data representation capabilities. The multiplication of complex values can represent the rotation and scale, corresponding to the angle addition and modular length multiplication of IQ data. While the multiplication of real values can only represent the scaling process, including only modulus multiplication. The IQ vector is converted from the original time series signal, which consists of two data vectors: in-phase components $x_{j}^{I}$ and quadrature components $x_{j}^{Q}$

(6) $$x_{j}^{I/Q}=\left[\begin{array}{c} x_{j}^{I^{T}}\\ x_{j}^{Q^{T}} \end{array}\right],$$

**Figure 3:**
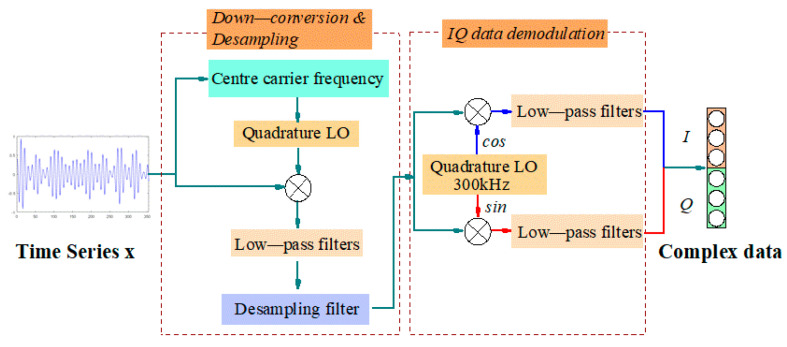
Time series signal converted into IQ data.

In (6), $x_{j}^{I^{T}},x_{j}^{Q^{T}}\in {\mathbb{R}}^{N}$, $x_{j}^{I/Q}\in {\mathbb{R}}^{2\times N}$. The process of converting the time series signal into IQ data is as follows: (1) multiply the original electromagnetic modulation signal and the carrier frequency to obtain the mixing result; (2) the down-conversion component of the mixed signal is obtained by low-pass filtering. The original time series modulated signal has a high carrier frequency and significant sampling points. Therefore, the purpose of reducing the sampling points and the computational complexity of the neural network is achieved by down-converting and down-sampling preprocessing of the original signal. The IQ data demodulated based on down-conversion and down-sampling time domain signals can be used as the input of CV-CNN for model training. The process of converting the time series signal into IQ data is shown in Figure 3.

### 2.3. Network Architecture

#### 2.3.1. The Motivation of the Network Made

**Figure 4:**
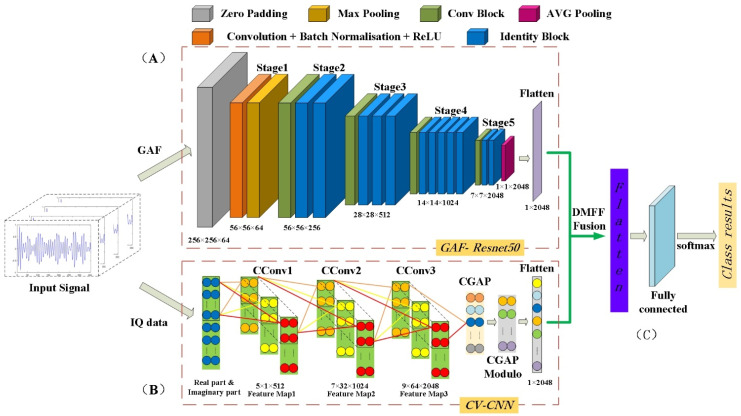
DMFF-CNN classification network structure. (**A**) The GAF-ResNet50 network structure; (**B**) The CV-CNN network structure; (**C**) Dual-modal feature fusion mechanism: DMFF.

In terms of feature extraction, the method based on deep learning can automatically extract higher-level features [39]. CV-CNN is similar to ResNet50, both use the feature extraction method from the bottom to the top to complete the classification of input data according to the in-depth features. However, the focus of the feature information retained by complex value CNN and two-dimensional CNN is different. The features extracted by ResNet50 highlight the local quality of the input image, while the CV-CNN makes full use of all the information of the IQ data. CV-CNN is a hierarchical link, including a complex input layer, multiple complex convolution layers and complex pooling layers, etc. Therefore, this paper uses GAF-ResNet50 and CV-CNN to extract features collaboratively and fuses dual-modal features. The fusion features go through the fully connected and softmax layers to achieve AMC through DMFF-CNN. Figure 4 shows the network structure of DMFF-CNN. In the following section, GAF-ResNet50, CV-CNN and features fusion will be introduced, respectively.

#### 2.3.2. GAF-ResNet50: Image Feature Extraction

Residual network (ResNet) proposes a residual learning method to reduce the difficulty of deep-seated training networks and solve the problem that image recognition accuracy decreases with the deepening of network training [40]. The ResNet structure is easy to optimize to obtain better performance. This paper builds the GAF-ResNet50 framework by restructuring the ResNet50 pretraining model to achieve the feature extraction of the GAF two-dimensional images. The structure is shown in Part (A) of Figure 4. The GAF-ResNet50 parameters and dimensions of each stage are shown in Table 1, and a detailed description of the architecture is explained below.

(1) Input layer

Convert the original modulation signal into a GAF image with a size of 256 × 256 and as network input. ResNet50 uses the convolution step of 2 × 2, and the number of channels is 64, so the input GAF image first compresses the height and width through the zero padding layer, the size of the feature layer becomes 112 × 112 × 64.

(2) Hidden layer

The hidden layer consists of Stage1~ Stage5 in Part (A) of Figure 4. The GAF image is Convolution (Conv), Batch-normalization (BN) and Activation_ReLU by Stage1, and then the height and width of the input feature layer are compressed to 56 × 56 × 64 through the maxpooling layer with a step size of 2 × 2. Stage2~ Stage5 contains two basic modules of ResNet50, namely Conv Block and Identity Block. Among them, the input and output characteristic dimensions of Conv Block are different, which plays a role in changing the dimension of GAF-ResNet50. The input and output dimensions of Identity Block are the same, which are used to deepen the number of layers of GAF-ResNet50. The Conv Block is divided into two parts: one is the backbone part, which has two Conv, BN, ReLU and one Conv, BN, and the other is the residual part, which has one Conv, BN. Such a structure is used to change the output feature layer’s width and height, and the number of channels. The Identity Block also has a backbone part and a residual part. The difference is that its residual part does not have a convolution operation and is directly connected to the output. Therefore, the shape of the input and output feature layers of the Identity Block is the same, which is used to deepen the network. This paper builds a deep network through the concatenated structure of Conv Block and Identity Block, adds the outputs of the backbone part and the residual part, and uses the Activation_ReLU to extract high-dimensional features of GAF images automatically.

(3) Output layer

The feature layer of 7 × 7 × 2048 is obtained by feature extraction from Stage2 to Stage5. Through AVG pooling, then complete the tiling of the feature layer of 1 × 1 × 2048, and finally output the feature vector with the length of 2048. In this paper, the network structure of ResNet50 is adjusted, and the last full connection layer is removed. The extracted features are tiled by adding an AVG pooling layer, which dramatically reduces the number of network parameters, makes the extracted GAF image features more intuitive and facilitates the fusion of dual-modal features by DMFF.

#### 2.3.3. CV-CNN: Complex Feature Extraction of IQ Data

This paper uses CV-CNN to extract deep complex features of IQ data. The network structure includes signal input layer, complex convolution layer (CConv), complex pooling layer (CGAP) and output layer. The structure of the network is shown in Part (B) of Figure 4. The CV-CNN parameters and dimensions of each frame are shown in Table 2 and the calculation details of the architecture are explained below.

(1) Input layer

The input of CV-CNN is IQ data. The feature maps and error terms generated by the forward and backward propagation of the network are in the form of complex values, and the parameter update is also performed in the complex value domain. A time window segments the IQ data with a length of 128. There are 120,000 samples in the complex value dataset.

(2) Complex Value convolution layer

The CV-CNN classification model consists of three convolution layers (CConv1, CConv2, CConv3). The dimensions of the convolution kernel in the three convolution layers are 5 × 1, 7 × 32 and 9 × 64. In order to make the extracted features more expressive, the number of convolution cores in each convolution layer is increased successively, which are 512, 1024 and 2048, respectively. Let $a_{k}^{\left(l-1\right)}\in C^{W_{l-1}\times H_{l-1}\times K}$ be the complex convolution layer input of layer l, where C represents the complex field and the dimension of $a_{k}^{\left(l-1\right)}$ is $W_{l-1}\times H_{l-1}\times K$. At the same time, if the *L-th* complex convolution layer contains I complex convolution cores $w_{ik}^{\left(l\right)}\in C^{F\times F\times K\times l}$, the characteristic maps of the output of the *L-th* complex convolution layer is $z_{i}^{\left(l\right)}\in C^{W_{l}\times H_{l}\times I}$, that is, the result of complex convolution of the input $a_{k}^{\left(l-1\right)}$ and $w_{ik}^{\left(l\right)}$. The calculation process is as (7)

(7) $$z_{i}^{\left(l\right)}=f\left(R\left(V_{i}^{\left(l\right)}\right)\right)+if\left(I\left(V_{i}^{\left(l\right)}\right)\right),$$

where f(·) represents the complex nonlinear activation function, and R(·) and I(·) mean the operation of taking the real part and imaginary part.$\;V_{i}^{\left(l\right)}$ represents the convolution result of the input feature maps $a_{k}^{\left(l-1\right)}$ and complex convolution cores $w_{ik}^{\left(l\right)}$. The convolution calculation process is shown in (8)

(8) $$\begin{array}{cc} V_{i}^{\left(l\right)} & =\overset{K}{\underset{k=1}{\sum }}w_{ik}^{\left(l\right)}\times a_{k}^{\left(l-1\right)}+b_{i}^{\left(l\right)}\\ & =\overset{K}{\underset{k=1}{\sum }}\left(R\left(w_{ik}^{\left(l\right)}\right)\times R\left(a_{ik}^{\left(l-1\right)}\right)-I\left(w_{ik}^{\left(l\right)}\right)\times I\left(a_{ik}^{\left(l-1\right)}\right)+R\left(b_{i}^{\left(l\right)}\right)\right)\\ & =i\overset{K}{\underset{k=1}{\sum }}\left(R\left(w_{ik}^{\left(l\right)}\right)\times I\left(a_{ik}^{\left(l-1\right)}\right)-I\left(w_{ik}^{\left(l\right)}\right)\times R\left(a_{ik}^{\left(l-1\right)}\right)+I\left(b_{i}^{\left(l\right)}\right)\right) \end{array},$$

The step size of the three-layer convolution is set to 1 without padding. The nonlinear complex activation function CReLU is used to increase the nonlinear expression ability of the network [41]. CReLU performs the ReLU operation on the real and imaginary parts of the IQ data. The specific calculation details are as follows

(9) CReLU(z)=ReLU(R(z))+i×ReLU(I(z))

A CPooling layer is added after each convolutional layer to reduce the features dimension and the number of parameters of the network. The pooling method adopts Maxpooling to increase the robustness of the modulation signal recognition network. The CBN layer is added after the first convolution layer to accelerate the convergence speed of CV-CNN. At the same time, the CDropout layer is introduced to reduce the overfitting phenomenon of the network, and the deactivation rate is set to 0.5. After three-layer convolution, the Feature Map3 in Part (B) of Figure 4 is integrated by accessing CV-CNN Global Average Pooling (CGAP). At this time, the feature is in complex form. The calculation details of the CPooling layer, CDropout layer and CGAP layer in the complex domain are shown in (10)~(12)

(10) CPooling(z)=Pooling(R(z))+i×Pooling(I(z))

(11) CDropout(z)=Dropout(R(z))+i×Dropout(I(z))

(12) CGAP(z)=GAP(R(z))+i×GAP(I(z))

(3) Output layer

Calculate the CGAP modulo of the output characteristic graph $z_{i}^{\left(l\right)}$ through (13)

(13) $$z^{\left(l+1\right)}=\sqrt{{\left(R\left(z^{l}\right)\right)}^{2}+{\left(I\left(z^{l}\right)\right)}^{2}},$$

where $z^{\left(l\right)}$ is the complex feature, and the modulus eigenvector $z^{\left(l+1\right)}$ of the complex feature is obtained. Finally, flatten $z^{\left(l+1\right)}$ to obtain a 1 × 2048 feature vector as the output of CV-CNN. Next, it is fused with the output features of GAF-ResNet50.

#### 2.3.4. Dual-Modal Feature Fusion Mechanism: DMFF

This paper proposes a DMFF mechanism to fuse the dual-modal features extracted by GAF-Resnet50 and CV-CNN. The structure is shown in Part (C) in Figure 4. The input of DMFF-CNN contains features from dual-modal, so the penalty between the two predicted label distributions needs to be considered. By adding penalty terms between each modal feature and connection feature, the classification accuracy and stability of the network can be improved. Jensen–Shannon (JS) divergence is used to calculate the penalty term. Let *p* and *q* represent the probability distribution of the GAF diagram and IQ data classification, respectively. JS divergence is defined as

(14) $$JS\left(p∥q\right)=\frac{1}{2}KL\left(p∥\frac{p+q}{2}\right)+\frac{1}{2}KL\left(q∥\frac{p+q}{2}\right),$$

Let $x_{i}^{m}$(m∈{1,2},i∈{1,⋯,N}) represent the *m-th* feature of the *i-th* data samples, and define $x_{i}^{c}$ as the fusion feature of the *i-th* data sample.$\;⊝\;=\left\{\theta^{c},\theta^{m}\right\}$ is obtained through model training, and the loss function of the classification model DMFF-CNN is expressed as (15)

(15) $$L\left(⊝\right)=\frac{1}{N}\sum_{i=1}^{N}JS\left(t_{i}∥p_{\theta^{c}}\left(x_{i}^{c}\right)\right)+\frac{\mu }{2}∥\theta^{c}∥{}^{2}+\frac{1}{N}\sum_{m=1}^{2}\sum_{i=1}^{N}JS\left(p_{\theta^{c}}\left(x_{i}^{c}\right)∥p_{\theta^{m}}\left(x_{i}^{m}\right)\right)+\frac{\mu }{2}∥\theta^{m}∥{}^{2},$$

In (15), $t_{i}$ represents the true probability distribution, μ is a hyperparameter, *N* represents the number of training samples, and the loss function includes a regularization term to avoid overfitting. The fusion process of the GAF diagram feature $x^{1}$ and IQ data feature $x^{2}$ continuously updates the parameters through the loss function θ until the result converges, and the final fusion feature $x^{c}$ is obtained. $x^{c}$ passes through a fully connected layer and softmax layer, and the classification probability distribution $p_{\theta }\left(x_{i}\right)$ of the network model is calculated, the operation process expression as (16):

(16) $$p_{\theta }\left(x_{i}\right)=softmax\left(z_{i}\right)=\frac{{\left[e^{\theta_{1}^{T}z_{i}},e^{\theta_{2}^{T}z_{i}},\cdots ,e^{\theta_{k}^{T}z_{i}}\right]}^{T}}{\sum_{k=1}^{K}e^{\theta_{k}^{T}z_{i}}},$$

In (16), the output result of the softmax layer is a vector of [1,K], where *K* is the classification type of the DMFF-CNN, and *K* = 8 is adopted in this paper.$\;z_{i}$ is the *i-th* value in the vector of the softmax layer, which is indexed by the softmax layer and batch normalized. With the increase in $z_{i}$, the change rate of $e^{\theta_{k}^{T}z_{i}}$ will be much higher than $z_{i}$. Therefore, the purpose of $z_{i}$ indexation is to make the maximum value in the input softmax layer more prominent. Meanwhile, make the prediction result of softmax layer output more significant, and improve the convergence efficiency of the model in training. In DMFF-CNN model prediction, p is defined as the expected label distribution. The probability distribution of the modulation signal proposed in this paper is calculated as (17) and (18)

(17) $$\underset{p}{\min}L\left(p|p_{\theta^{1}},p_{\theta^{2}}\right)=\sum_{m=1}^{2}\sum_{k=1}^{8}p\left(k\right)ln\left(\frac{p\left(k\right)}{p_{\theta^{m}}\left(k\right)}\right),$$

(18) $$s.t.\sum_{k=1}^{8}p\left(k\right)=1,$$

Finally, the Lagrange function is constructed by (19), and the classification probability of the model for various types of modulation signals can be obtained.

(19) $$\;p\left(k\right)=\frac{\sqrt{\prod_{m}p_{\theta^{m}}\left(k\right)}}{\sum_{j}^{8}\sqrt{\prod_{m}p_{\theta^{m}}\left(j\right)}},$$

## 3. Experiments and Results

### 3.1. Parameter Settings and Datasets Description

Traditional datasets based on DL methods (such as rml2016.10a) lack generalization ability in construction. Considering the characteristics of abnormal electromagnetic signals in the actual environment, this paper filters and expands the signal types of the existing datasets. The dataset is constructed according to the parameters in Table 3, including eight types of electromagnetic signal samples of digital modulation and analog modulation (2FSK, AM, DSB, FM, OFDM, QAM16, QPSK, SSB). The signal parameters include chip rate (CR), carrier frequency, modulation frequency and phase difference. The SNR is set to −10 dB to 10 dB, and the step size is 2 dB. The signal is assumed to be acquired in the environment disturbed by Additive White Gaussian Noise (AWGN). SNR is defined as $\mathrm{SNR}=10log_{10}\left(\sigma_{s}^{2}\right)/\left(\sigma_{\varepsilon }^{2}\right)$, where $\sigma_{s}^{2}$ is the signal variance and $\sigma_{\varepsilon }^{2}$ is the variance of AWGN. The signal sampling frequency is 102 MHz, the sampling time of each group is 10 ms, the IF sampling frequency is 3.2 MHz and the target frequency of down-conversion is 300 KHz. All kinds of modulated signals generate 1500 samples under each SNR situation. The number of signal samples in the datasets is 120,000, of which 70% are used as training samples and 30% are verification samples. The parameters of the multi-type modulation signals are shown in Table 3.

### 3.2. The Result of the Datasets Preprocessing

**Figure 5:**
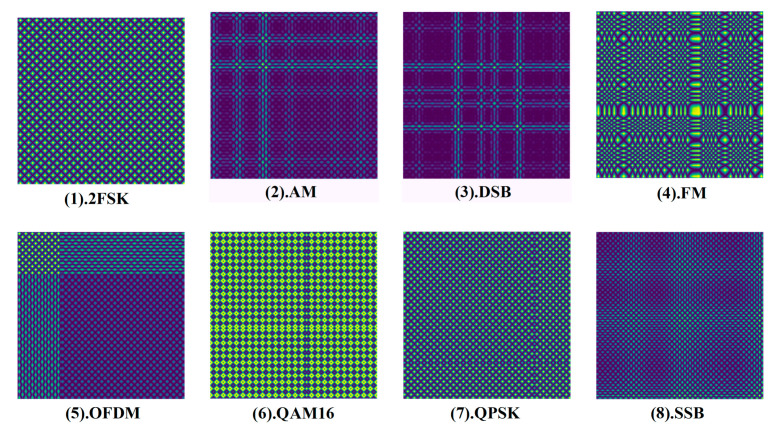
Two-dimensionalization of eight types of modulated signal-based GAF.

**Figure 6:**
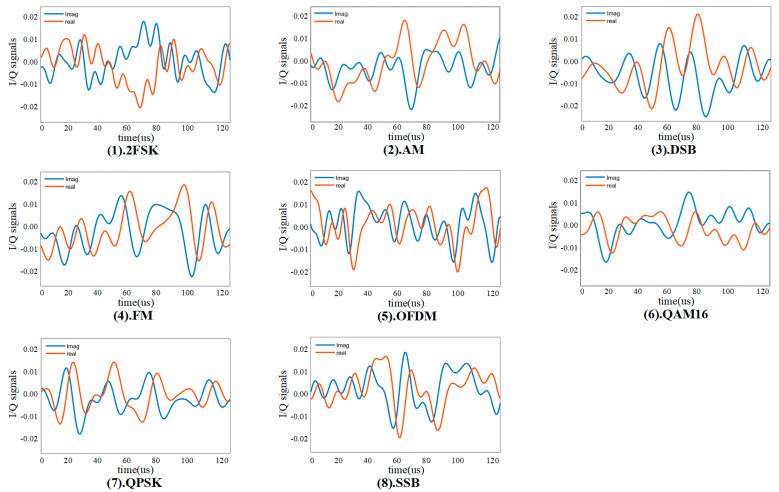
IQ sequences converted from eight types of modulated signals.

According to the signal preprocessing method in Section 2, the eight types of one-dimensional electromagnetic modulation signal samples in Table 3 are processed, respectively. Based on GAF theory, the time series signal is mapped to 2D images, the image size is 256 × 256 and the resolution is 300 dpi, which meets the input format of the GAF-ResNet50. The results with SNR of 5 dB are selected for display, as shown in (1–8) in Figure 5. We can find that there are apparent differences in the color features of 2D images generated by GAF for different types of signals, which is beneficial to GAF-ResNet50 for deep feature extraction. At the same time, the time series signal is converted into IQ data, and the signal is segmented through a rectangular window. Each segment of data consists of in-phase sequence and quadrature sequence. Figure 6 (1–8) show the IQ sequences converted from eight types of modulated signals used as the input of CV-CNN for feature extraction.

### 3.3. Experimental Results and Evaluation

This paper’s data generation and processing are based on matlab2021b and python 3.9. The classification model training experimental environment is based on Tensorflow 3.0 on Intel (R) Gold 5188 CPU and NVIDIA Quadro P400 GPU.

#### 3.3.1. Training Results of Three Classification Models

The classification accuracy training of GAF-Resnet50, CV-CNN and DMFF-CNN are discussed.

(1) The AMC of the modulation signal based on GAF combines GAF-ResNet50 and the softmax layer for feature extraction and classification recognition. The training process mainly updates the weight parameters of the CNN. The classifier obtains the modulation classification results and completes the backpropagation.
(2) The AMC based on IQ data combines the CV-CNN and softmax layer. The training process mainly updates the CV-CNN weight parameters. The classifier obtains the modulation recognition results and completes the backpropagation.
(3) The input of the DMFF-CNN is obtained by fusing the output feature vectors of the above two classification models through DMFF. After passing through the full connection layer, the fused features are input into the softmax classifier. Unlike GAF-ResNet50 and the CV-CNN, the DMFF-CNN training process only trains the softmax classifier. The learning rate of the above three classification network models is 0.0005, the batch size is 64 and the epochs is 90.

**Figure 7:**
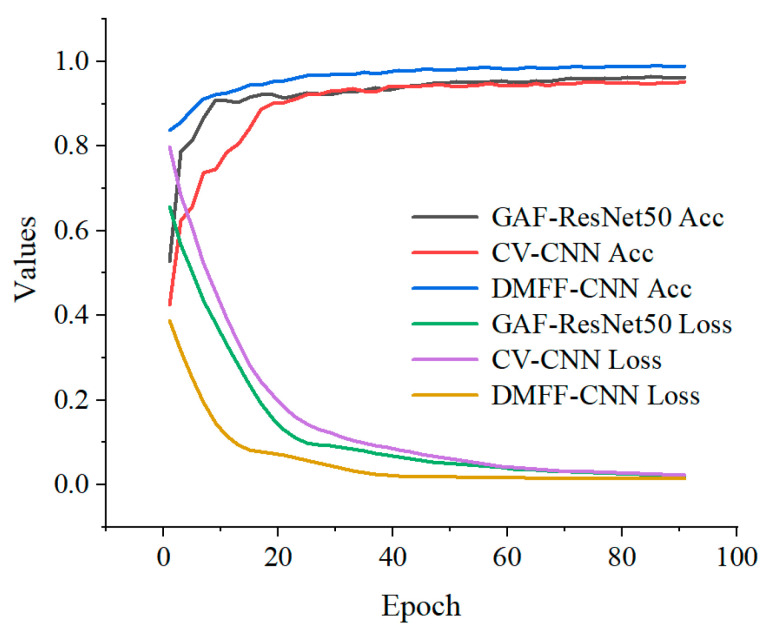
Training results of three classification models.

The loss value and ACC indicators evaluate the performance of the three classification models, and the results are shown in Figure 7. It can be concluded that when the ACC value rises and the loss value decreases and tends to be stable, the DMFF-CNN proposed in this paper is better than the other two classification models, which proves the performance of the classification model can be improved based on the DMFF mechanism. The CV-CNN performs the worst among the three classification models. The reason may be that the dimension of IQ data is much lower than the GAF image.

**Figure 8:**
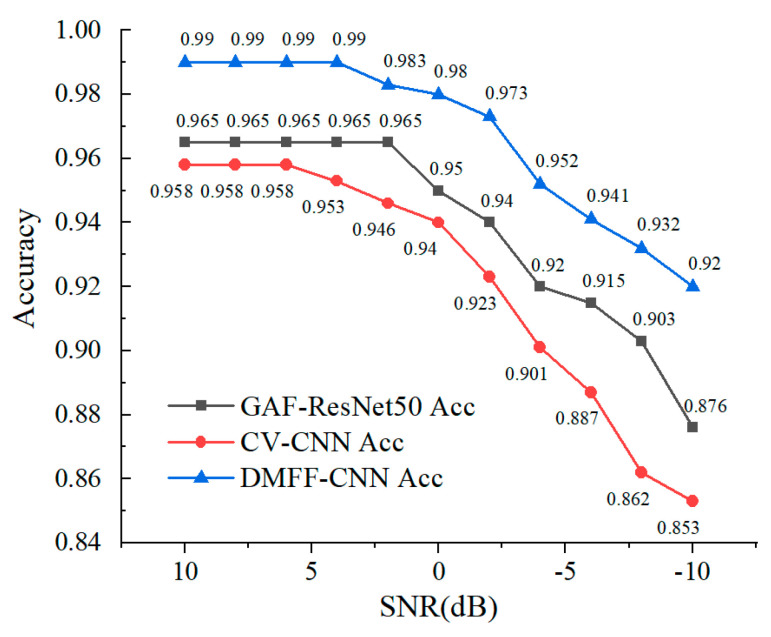
The average recognition accuracy of three models under different SNR.

To further illustrate the advantages of the DMFF-CNN, the average recognition accuracy of the three models under different SNR situations is tested. The experimental results are shown in Figure 8.

It can be seen from Figure 8 that with the improvement in SNR, the classification accuracy of the three models is significantly improved. In the case of deteriorating SNR, the classification accuracy of the DMFF-CNN is better than the other two networks and can reach 92.1% with SNR at −10 dB. Among them, the recognition accuracy of GAF-Resnet50 is 87.6%, which is in the middle, while the recognition accuracy of the CV-CNN is poor, only 85.3%.

**Figure 9:**
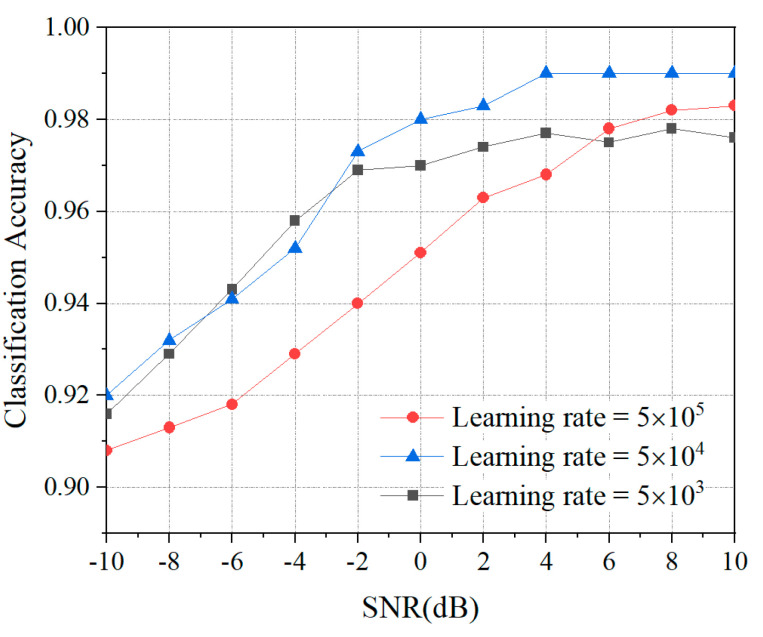
Classification accuracy of DMFF-CNN under different learning rates and SNR.

Considering the learning rate parameters in the training process of the DMFF-CNN, and in order to maximize the classification accuracy and optimize the training time, the learning rate is adjusted and the impact on the accuracy of AMC is recorded. The results are shown in Figure 9.

The DMFF-CNN has the best classification accuracy at a learning rate of 0.0005, and it drops at 0.005 and 0.00005. This is because the low learning accuracy requires a high number of epochs for the classification model, which leads to the slow convergence speed of the model and the inability to achieve the optimal solution in the limited number of epochs. Conversely, a high learning rate will lead to the rapid convergence of the network model, resulting in the optimal solution being ignored, especially in the case of low SNR situations.

#### 3.3.2. Classification Accuracy of Three Models under Different SNR

**Figure 10:**
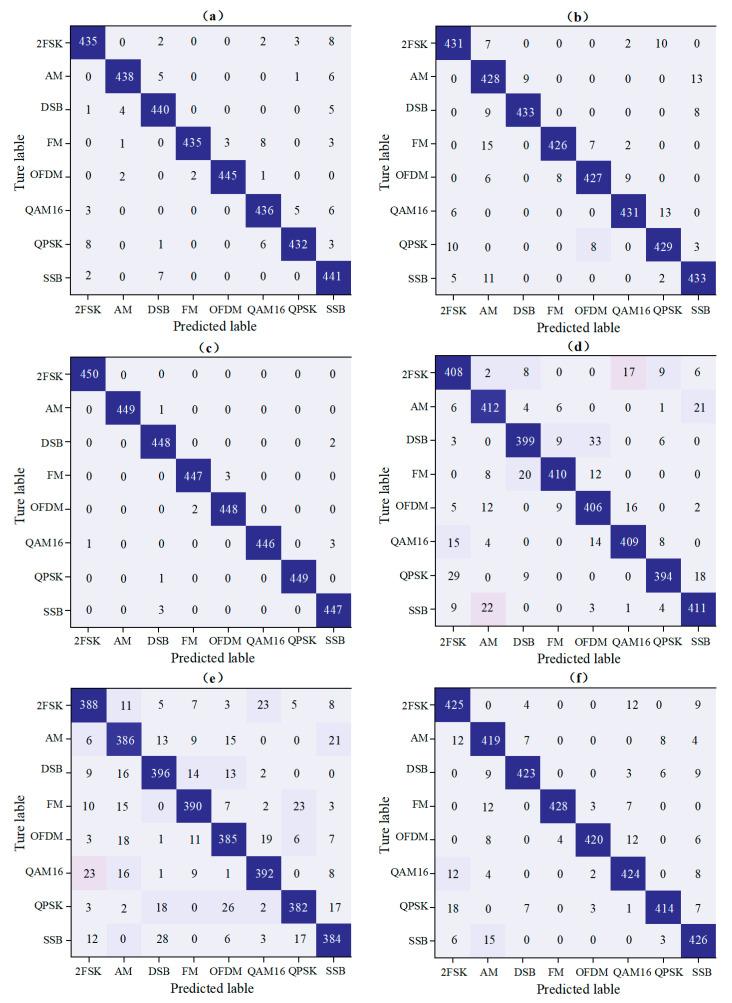
The average classification accuracy confusion matrix of GAF-ResNet50, CV-CNN and DMFF-CNN models under high SNR interval (4~10 dB) and low SNR interval (−4~10 dB). (**a**–**c**) correspond to the three models at high SNR interval; (**d**–**f**), respectively, correspond to the three models at low SNR interval.

In order to study the factors that limit the AMC accuracy of the three network models under different SNR, the SNR interval is divided into high SNR interval (4~10 dB) and low SNR interval (−4~10 dB). The classification accuracy of the three models is tested by using validation samples. The recognition accuracy in the two SNR intervals is averaged to obtain the confusion matrix, which is the average classification accuracy results of the three classification network models. The results are shown in Figure 10.

The prediction results of the three classification network models for various types of modulation signals can be seen intuitively in Figure 10. Each column of the confusion matrix represents the real category, and each row represents the prediction category. The results show that the DMFF-CNN classification model has high discrimination accuracy for all types of signals and excellent robustness in a low SNR environment. The advantages of DMFF-CNN are mainly reflected in two aspects: One is that GAF-ResNet50 and the CV-CNN realize complementary advantages in dual-modal feature extraction; the other is the role of the DMFF feature fusion mechanism.

**Figure 11:**
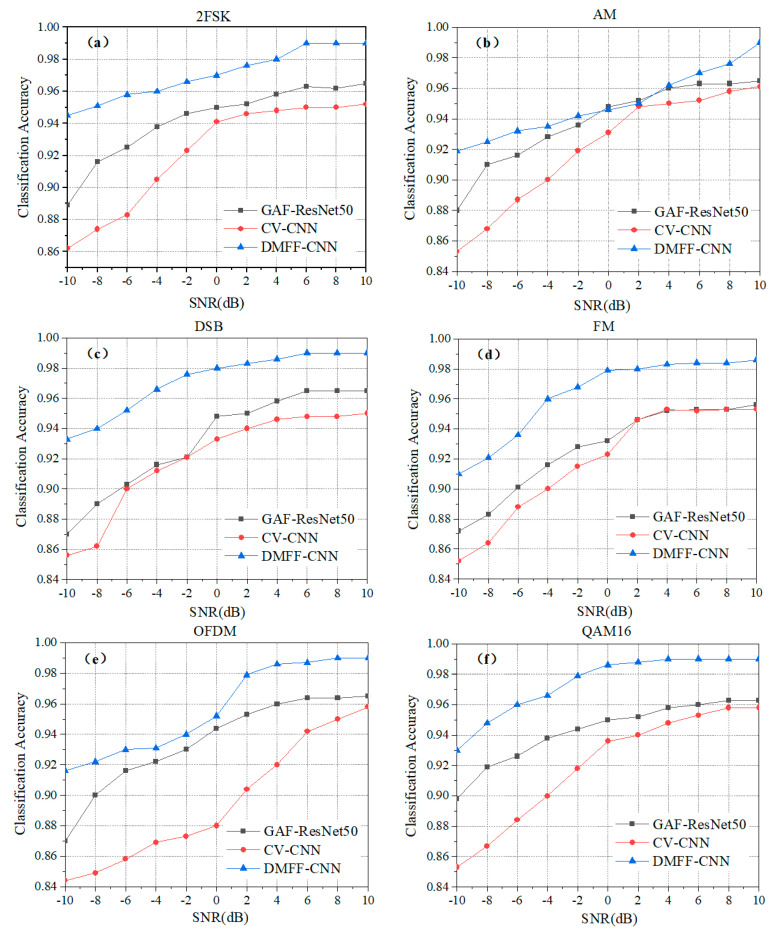

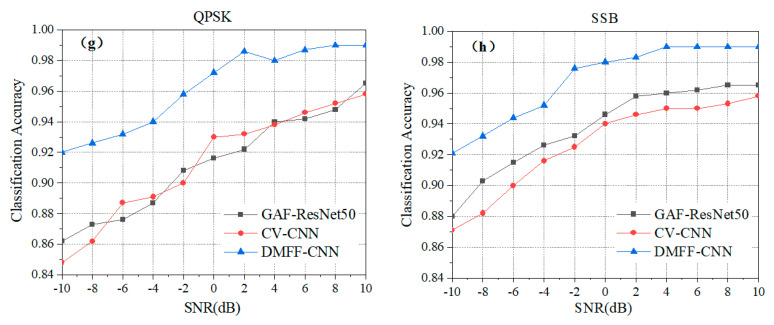
The classification accuracy of each type of modulated signal in the three models varies with SNR. (**a**) 2FSK; (**b**) AM; (**c**) DSB; (**d**) FM; (**e**) OFDM; (**f**) QAM16; (**g**) QPSK; (**h**) SSB.

Figure 11 plots the classification accuracy of each modulation type under the three classification models to obtain the performance of a specific modulation type that varies with SNR. The DMFF-CNN model proposed in this paper is better than the other two, especially at low SNR. Considering the poor signal transmission conditions in the actual communication environment, it is more meaningful to have high classification accuracy at the state of low SNR. In the modulated signal datasets, QAM16 has the highest accuracy, as shown in Figure 11f, from which it can be understood that QAM16 contains multiple frequency components. It can also be found that GAF-ResNet50 performs better than the CV-CNN in various types of signal classification, especially in 2FSK, OFDM, QAM16 and SSB. The reason is that a GAF image contains higher dimensional information than IQ data, which is reflected in the high-level feature extraction of neural networks. The above results further reflect the advantages of GAF in the characterization of modulated signals.

## 4. Discussion

In this section, a series of comparative experiments are carried out with the eight types of electromagnetic modulation signals shown in Table 3 to evaluate the performance of our method. Firstly, the methods of using Single-Feature for AMC are analyzed and compared with the dual-modal fusion method proposed in this paper, which proves the effectiveness of the feature fusion mechanism (DMFF). Furthermore, comparing with AMC methods using different feature combinations, the results are discussed with our method, which demonstrates the advantages of feature fusion using GAF images and IQ sequences.

### 4.1. Advantages of Dual-Modal Feature Fusion Mechanism: DMFF

In the AMC methods based on DL, the Single-Feature representation is mostly used. There are three representative categories: Feature representation [21], Sequence representation [29,42] and Image representation [23,25]. In the low SNR environment of −10 dB, combined with the dual-modal feature (GAF images and IQ sequence) method, the average classification accuracy of the dataset is shown in Table 4.

Table 4 shows that the DMFF mechanism proposed in this paper has obvious advantages over the Single-Feature AMC methods. The shortcomings of the Feature representation method [21] are obvious. Compared with other Single-Feature classification methods, the reasons for the poor effect are as follows: Firstly, the method of extracting features directly from the original signal leads to a large increase in computational complexity and a sharp drop in performance in low SNR environments. Secondly, feature selection depends on human experience, which leads to poor applicability in different signal classification tasks. Finally, for multi-type electromagnetic modulation signals, the incomplete characterization easily leads to the loss of key information in the original signal. It is worth mentioning that the method in this paper has a 60.1% improvement compared to Spectral Features.

The method’s performance based on Sequence representation b, [29,42] is at the middle level. Although the Sequence representation method takes advantage of electromagnetic signal samples that are sequentially received, the amount of calculation is small. However, the CV-CNN proposed in this paper combines the characteristics of IQ data to extract complex features of the signal, and the classification effect is better than [29,42]. Therefore, the method of Sequence representation requires an actual and reasonable CNN network according to the signal characteristics. If the network structure is poor, converging is not easy. At the same time, the performance of this method declines in low SNR, so different representation methods need to be selected according to the noise environment.

Image representation-based methods a, [23,25] represent the received signal as images and combine the DL framework to achieve automatic feature extraction. Converting sequential signal recognition to a 2D image has better performance than Feature representation and Sequence representation methods. Compared with a, [23,25], the feature fusion method proposed in this paper is improved by 4.5%, 5.3% and 9.8%, respectively. However, using the single-image method alone also has limitations as image layered information requires a deeper and more complex CNN to achieve feature extraction tasks.

To avoid the above problems, a dual-modal feature fusion mechanism is designed to avoid the defects of single-feature representation. GAF image and IQ data feature extraction are achieved by GAF-ResNet50 and CV-CNN, respectively, by adding penalty terms between dual-modal features to reduce the complexity of the network, and Jensen–Shannon being used to map the classification problem to a probability problem. At the same time, in DMFF-CNN training, a regularization term to avoid overfitting is added to the loss function to improve the convergence rate of the classification model in the training process. By fusing GAF image and IQ sequence features, the complementary advantages of different modal features of the modulation signal are realized.

### 4.2. Experimental Results and Evaluation

Many research achievements have been made in representing the original modulated signal through the combination of different signal features [30,31,32,33,34,35,36]. Table 5 lists some state-of-the-art methods for AMC by fusing different modal features. The comparative experiment is carried out within the SNR range of (−10 dB, 10 dB), and the experimental modulation signals are affected by AWGN. Table 5 records the average classification accuracy of various methods on the signals of the dataset.

**Figure 12:**
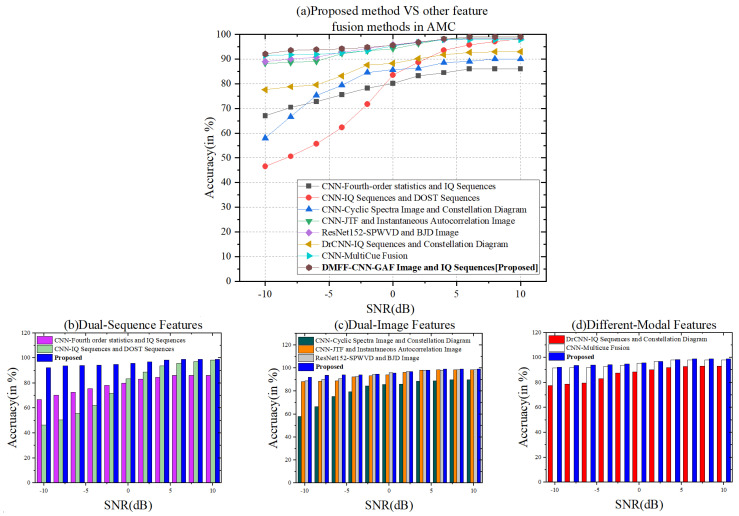
(**a**) The relationship between the average classification accuracy and SNR range of methods in Table 5; (**b**) the comparison results of the classification accuracy using the dual-sequence features fusion methods vs DMFF-CNN; (**c**) the comparison results of the classification accuracy using the dual-image features fusion methods vs DMFF-CNN; (**d**) the comparison results of the classification accuracy using the different modal features fusion methods vs DMFF-CNN.

The modulation classification algorithm based on DL in Table 5 represents the original signal in various formats. It is clear that the original modulation signal is represented by the combination of multiple features, images or sequences, which can integrate the advantages of various features and obtain better classification performance. Figure 12a shows the relationship between the average classification accuracy and SNR of different methods in Table 5. Meanwhile, to explain the influence of varying feature fusion methods, the eight methods included in Table 5 are divided into three categories: dual-sequence features [34,36], dual-image features [30,32,33] and different modal features [35,43] and the proposed. Figure 12b–d show the comparison of classification accuracy between the three fusion categories and the methods presented in this paper.

By contrast, in the case of low SNR, the classification methods using different modal features fusion has obvious advantages. We can clearly observe from Figure 12b–d where the dual-sequence features fusion method performs worst, which also verifies the conclusions obtained in Table 4. It also shows that the use of uncorrelated and different modal combined features will improve the performance of AMC. The classification accuracy of image-feature fusion methods [30,32,33] is better than that of sequence feature fusion methods [34,36]. Combined with the analysis results in Table 5, it can be seen that the dimension of the sequence feature (1D) is much lower than the image feature (2D). The image feature fusion method [33] combined with ResNet-152 has better classification performance than [30,32] (increased by 31% and 0.7%, respectively) at low SNR. Therefore, it can be considered that a deeper network structure will lead to better classification performance.

Furthermore, the factors that limit the average classification accuracy of modulated signals under low SNR conditions are further explored. The methods in references [32,33] all adopt image-feature combination and achieve good classification results, and the classification accuracy reaches 88.3% and 89% under low SNR (-10 dB). Compared with the above two methods, the dual-modal feature fusion method proposed in this paper improves by 3.8% and 3.1%, respectively. The reason for the analysis is that the classification method of JTF image and instantaneous autocorrelation image combined with CNN is adopted in [32], but JTF image is not sensitive to amplitude and phase modulation signals, and instantaneous autocorrelation images easily confuse the frequency coded signals. However, there are AM and FM signal types in the dataset mentioned in Table 3, so the classification accuracy is affected. In the method of [33], SPWVD and BJD images are used for modulation signal characterization. Still, the disadvantage is that there is no corresponding classification network designed for different types of images. Conversely, [33] used the same CNN network structure for feature extraction, causing the same kind of and multiple feature defects to accumulate and play a dominant role, affecting the classification performance.

To sum up, the feature combination needs to be reasonably selected according to the fusion mechanism for AMC. At the same time, it is imperative to build a suitable network model. The DMFF-CNN proposed in this paper combines the features of dual-modal with significant differences and complementarity. Compared with other advanced methods, it shows good robustness in different SNR environments and superior performance in AMC classification accuracy.

## 5. Conclusions

The focus of this paper is to solve the problem of AMC methods ignoring the complementarity and feature fusion between different modal features in feature selection, and we propose a new classification model based on the dual-modal feature fusion convolutional neural network (DMFF-CNN). GAF image coding theory is introduced into the field of AMC for the first time, by converting the received original modulation signal into GAF image and IQ data, respectively. The network structure of GAF-ResNet50 and CV-CNN is further optimized to realize the feature extraction of data with a dual-modal approach. Most importantly, a dual-modal feature fusion mechanism (DMFF) is proposed, and the DMFF-CNN is trained through the dual-modal fusion features. The experimental verification is carried out through the datasets of eight kinds of modulation signals. Finally, the experiment results show that the DMFF-CNN classification model proposed in this paper achieves an accuracy of 92.1% in the SNR environment of −10 dB, which further indicates that this method has good robustness for AMC in the harsh communication environment. Based on theory and experiment, we draw the following conclusion.

- Dual-modal feature fusion CNN makes full use of the complementarity between different modal features, gram angular field (GAF) images and IQ data combined with DMFF-CNN demonstrate excellent AMC performance. Therefore, using the different advantages of images and time series in signal representation, combined with a suitable fusion mechanism, will greatly improve the performance of AMC.
- It will be of great value to research the impacts of representations on modulated signals. Different representations retain different received signal characteristics, as with two-dimensional images converted by GAF and one-dimensional IQ data. In addition, since there are advantages of different networks in handling different types of signals, an appropriate network structure should be designed to consider the signal representation fully. In this way, the advantages of different networks can be fully utilized and combined.

In further works, we will focus on the following two aspects. Firstly, to improve the generalization and applicability of the proposed network model and electromagnetic modulation signal classification method, we will try to fuse the features of higher-order and more types of modulation signals, and carry out classification and recognition experiments. In addition, this paper considers dual-modal feature fusion and combines neural networks for AMC, which can lead us to further explore multi-modal and multi-network feature fusion methods. Solving the AMC problem is the focus of our work by taking into account the operation speed and classification effect.

## Figures and Tables

**Table 1:** Detailed parameters and dimensions of the GAF-ResNet50.

Stages	Layers Types	Activation	Kernel Size/Strides	Output Size
0	Input image	-	-	256 × 256 × 3
	Zero padding	-	-	
1	Convolution	ReLU	7 × 7 × 64/2	112 × 112 × 64
	Batch_Normaliz	-	-	56 × 56 × 64
	Maxpooling	-	3 × 3 × 64/2	
2	Conv Block×1	ReLU	64 × 64 × 256/1	56 × 56 × 256
	Identity Block×2	ReLU	64 × 64 × 256/-	
3	Conv Block×1	ReLU	128 × 128 × 512/1	28 × 28 × 512
	Identity Block×3	ReLU	128 × 128 × 512/-	
4	Conv Block×1	ReLU	256 × 256 × 1024/1	14 × 14 × 1024
	Identity Block×5	ReLU	256 × 256 × 1024/-	
5	Conv Block×1	ReLU	512 × 512 × 2048/1	7 × 7 × 2048
	Identity Block×2	ReLU	512 × 512 × 2048/-	
6	AVG pooling	-	7 × 7/2	1 × 1 × 2048
7	Flatten	-	-	1 × 2048

**Table 2:** Detailed parameters and dimensions of the CV-CNN.

Frames	Feature Types	Activation	Pooling	Batch_Normaliz	Dropout	Output Size
Input layers	Real part and Imaginary part	-	-	-	-	128
CConv1	Feature Map1	CReLU	MaxPooling	CBN	-	5 × 1 × 512
CConv2	Feature Map2	CReLU	MaxPooling	-	-	7 × 32 × 1024
CConv3	Feature Map3	CReLU	MaxPooling	-	50%	9 × 64 × 2048
CGAP	-	-	-	-	-	1 × 1 × 2048
CGAP Modulo Calculation						
Flatten	-	-	-	-	-	1 × 2048

**Table 3:** Sample parameters of the electromagnetic modulation signal.

	2FSK	AM	DSB	FM	SSB	QAM16	QPSK	OFDM
CR (kHz)	2~20	/				2~20		
Carrier Frequency (MHz)	$\begin{array}{c} f_{1}:1.5\sim 30\\ f_{2}:f_{1}+\left(10\sim 80\right) \end{array}$	1.5~30						
Modulation frequency (kHz)	/	f_1_ = 1~3 f_2_ = 3~5 f_3_ = 5~7 f_4_ = 7~9 f_5_ = 9~11				/		
Amplitude (V)	0.25~1							

**Table 4:** Comparison of accuracy between DMFF and other Single-Feature methods in AMC. (Bold is the best experimental result).

Feature	GAF Image (a)	IQ Sequences (b)	Spectral Features [21]	IQ Sequence [42]
Network	GAF-ResNet50	CV-CNN	SAE-DNN	CNN
SNR (dB)	−10	−10	−10	−10
Average Accuracy	87.6% (↑ 4.5%)	85.3% (↑ 6.8%)	32% (↑ 60.1%)	65% (↑ 27.1%)
Min Accuracy	86.2% (↑ 4.8%)	84.4% (↑ 6.6%)	31.3% (↑ 59.7%)	64.2% (↑ 26.8%)
Max Accuracy	88.9% (↑ 5.6%)	87% (↑ 7.5%)	33.1% (↑ 61.4%)	65.3% (↑ 29.2%)
Feature	Constellation Density Matrix [23]	Cyclic Correntropy spectrum Graph [25]	FFT Sequence [29]	GAF and IQ Sequences (Proposed)
Network	ResNet50	Deep-ResNet	MTL-CNN	DMFF-CNN
SNR (dB)	−10	−10	−10	−10
Average Accuracy	86.8% (↑ 5.3%)	82.3% (↑ 9.8%)	59.4% (↑ 32.7%)	**92.1%**
Min Accuracy	84.9% (↑ 6.1%)	80.8% (↑ 10.2%)	57.6% (↑ 33.4%)	**91%**
Max Accuracy	87.6% (↑ 6.9%)	83.5% (↑ 11%)	62.1% (↑ 32.4%)	**94.5%**

**Table 5:** Comparison of the method’s accuracy proposed in this paper and other feature fusion methods in AMC.( Bold is the best experimental result.)

Method	Fourth-Order Cumulants and IQ Sequences [34]	IQ Sequences and Constellation Diagram [35]	Cyclic Spectra Image and Constellation Diagram [30]	JTF Image and Instantaneous Autocorrelation Image [32]
Network	CNN and LSTM	DrCNN	CNN	CNN
Accuracy	LSTM: 39–83% CNN: 67–86%	77.6–93%	58–90%	88.3–98.6%
Method	SPWVD and BJD Image [33]	IQ Sequences and DOST Sequences [36]	Multi-Cue Fusion [43]	GAF and IQ Sequences (Proposed)
Network	ResNet-152	CNN	CNN	**DMFF-CNN**
Accuracy	89–98.5%	46.5–98.3%	91.5–97.9%	**92.1–99%**

## Data Availability

All modulation data and code will be made available on request to the correspondent author’s email with appropriate justification.

## References

[1] Dobre O., Abdi A., Bar-Ness Y., Su W. (2007). Survey of automatic modulation classification techniques: Classical approaches and new trends. IET Commun..

[2] Hakimi S., Hodtani G.A. (2017). Optimized Distributed Automatic Modulation Classification in Wireless Sensor Networks Using Information Theoretic Measures. IEEE Sens. J..

[3] Meng F., Chen P., Wu L., Wang X. (2018). Automatic Modulation Classification: A Deep Learning Enabled Approach. IEEE Trans. Veh. Technol..

[4] Xu J.L., Su W., Zhou M. (2010). Likelihood-Ratio Approaches to Automatic Modulation Classification. IEEE Trans. Syst. Man Cybern. Part C (Appl. Rev.).

[5] Han L., Gao F., Li Z., Dobre O.A. (2017). Low complexity automatic modulation classification based on order-statistics. IEEE Trans. Wirel. Commun..

[6] Dobre O., Öner M., Rajan S., Inkol R. (2011). Cyclostationarity-Based Robust Algorithms for QAM Signal Identification. IEEE Commun. Lett..

[7] Pawar S.U., Doherty J.F. (2011). Modulation Recognition in Continuous Phase Modulation Using Approximate Entropy. IEEE Trans. Inf. Forensics Secur..

[8] Deng Y., Wang Z. (2014). Modulation recognition of MAPSK signals using template matching. Electron. Lett..

[9] Kharbech S., Dayoub I., Zwingelstein-Colin M., Simon E.P. (2016). On classifiers for blind feature-based automatic modulation classification over multiple-input–multiple-output channels. IET Commun..

[10] Xie L., Wan Q. (2017). Automatic Modulation Recognition for Phase Shift Keying Signals With Compressive Measurements. IEEE Wirel. Commun. Lett..

[11] Mao Q., Hu F., Hao Q. (2018). Deep Learning for Intelligent Wireless Networks: A Comprehensive Survey. IEEE Commun. Surv. Tutor..

[12] Tu Y., Lin Y. (2019). Deep Neural Network Compression Technique Towards Efficient Digital Signal Modulation Recognition in Edge Device. IEEE Access.

[13] Neshat M., Nezhad M.M., Abbasnejad E., Mirjalili S., Tjernberg L.B., Garcia D.A., Alexander B., Wagner M. (2021). A deep learning-based evolutionary model for short-term wind speed forecasting: A case study of the Lillgrund offshore wind farm. Energy Convers. Manag..

[14] Kwon D.-H., Kim J.-B., Heo J.-S., Kim C.-M., Han Y.-H. (2019). Time series classification of cryptocurrency price trend based on a recurrent LSTM neural network. J. Inf. Processing Syst..

[15] Koutsoukas A., Monaghan K.J., Li X., Huan J. (2017). Deep-learning: Investigating deep neural networks hyper-parameters and comparison of performance to shallow methods for modeling bioactivity data. J. Cheminform..

[16] Pareek V., Chaudhury S. (2021). Deep learning-based gas identification and quantification with auto-tuning of hyper-parameters. Soft Comput..

[17] Peng S., Sun S., Yao Y.D. (2021). A Survey of Modulation Classification Using Deep Learning: Signal Representation and Data Preprocessing. IEEE Trans. Neural Netw. Learn. Syst..

[18] Ali A., Yangyu F. (2017). Automatic Modulation Classification Using Deep Learning Based on Sparse Autoencoders With Nonnegativity Constraints. IEEE Signal Process. Lett..

[19] Xie W., Hu S., Yu C., Zhu P., Peng X., Ouyang J. (2019). Deep Learning in Digital Modulation Recognition Using High Order Cumulants. IEEE Access.

[20] Lee S.H., Kim K.-Y., Kim J.H., Shin Y. Effective Feature-Based Automatic Modulation Classification Method Using DNN Algorithm. Proceedings of the 2019 International Conference on Artificial Intelligence in Information and Communication (ICAIIC).

[21] Shah M.H., Dang X. (2019). Classification of Spectrally Efficient Constant Envelope Modulations Based on Radial Basis Function Network and Deep Learning. IEEE Commun. Lett..

[22] Shi W., Liu D., Cheng X., Li Y., Zhao Y. (2019). Particle Swarm Optimization-Based Deep Neural Network for Digital Modulation Recognition. IEEE Access.

[23] Kumar Y., Sheoran M., Jajoo G., Yadav S.K. (2020). Automatic Modulation Classification Based on Constellation Density Using Deep Learning. IEEE Commun. Lett..

[24] Wang D., Zhang M., Li Z., Li J., Fu M., Cui Y., Chen X. (2017). Modulation Format Recognition and OSNR Estimation Using CNN-Based Deep Learning. IEEE Photon-Technol. Lett..

[25] Ma J., Lin S.-C., Gao H., Qiu T. Automatic Modulation Classification Under Non-Gaussian Noise: A Deep Residual Learning Approach. Proceedings of the ICC 2019—2019 IEEE International Conference on Communications (ICC).

[26] Li Y., Shao G., Wang B. Automatic Modulation Classification Based on Bispectrum and CNN. Proceedings of the 2019 IEEE 8th Joint International Information Technology and Artificial Intelligence Conference (ITAIC).

[27] Huang L., Pan W., Zhang Y., Qian L., Gao N., Wu Y. (2019). Data Augmentation for Deep Learning-Based Radio Modulation Classification. IEEE Access.

[28] Hermawan A.P., Ginanjar R.R., Kim D.-S., Lee J.-M. (2020). CNN-Based Automatic Modulation Classification for Beyond 5G Communications. IEEE Commun. Lett..

[29] Mossad O.S., ElNainay M., Torki M. Deep Convolutional Neural Network with Multi-Task Learning Scheme for Modulations Recognition. Proceedings of the 2019 15th International Wireless Communications & Mobile Computing Conference (IWCMC).

[30] Wu H., Li Y., Zhou L., Meng J. (2019). Convolutional neural network and multi-feature fusion for automatic modulation classification. Electron. Lett..

[31] Mao Y., Dong Y.-Y., Sun T., Rao X., Dong C.-X. (2021). Attentive Siamese Networks for Automatic Modulation Classification Based on Multitiming Constellation Diagrams. IEEE Trans. Neural Netw. Learn. Syst..

[32] Wang F., Yang C., Huang S., Wang H. (2019). Automatic modulation classification based on joint feature map and convolutional neural network. IET Radar, Sonar Navig..

[33] Zhang Z., Wang C., Gan C., Sun S., Wang M. (2019). Automatic Modulation Classification Using Convolutional Neural Network With Features Fusion of SPWVD and BJD. IEEE Trans. Signal Inf. Process. Over Netw..

[34] Zhang M., Zeng Y., Han Z., Gong Y. Automatic Modulation Recognition Using Deep Learning Architectures. Proceedings of the 2018 IEEE 19th International Workshop on Signal Processing Advances in Wireless Communications (SPAWC).

[35] Wang Y., Liu M., Yang J., Gui G. (2019). Data-Driven Deep Learning for Automatic Modulation Recognition in Cognitive Radios. IEEE Trans. Veh. Technol..

[36] Hiremath S.M., Behura S., Kedia S., Deshmukh S., Patra S.K. Deep Learning-Based Modulation Classification Using Time and Stockwell Domain Channeling. Proceedings of the 2019 National Conference on Communications (NCC).

[37] Wang Z., Oates T. Encoding Time Series as Images for Visual Inspection and Classification Using Tiled Convolutional Neural Networks. Proceedings of the Workshops at the Twenty-Ninth Aaai Conference on Artificial Intelligence.

[38] Wang Z., Oates T. (2015). Imaging time-series to improve classification and imputation. Proceedings of the 24th International Conference on Artificial Intelligence.

[39] Zhang Z., Wang L., Zou Y., Gan C. (2018). The optimally designed dynamic memory networks for targeted sentiment classification. Neurocomputing.

[40] He K., Zhang X., Ren S., Sun J. Deep residual learning for image recognition. Proceedings of the 2016 IEEE Conference on Computer Vision and Pattern Recognition (CVPR).

[41] Zhang H., Yu L., Chen Y., Wei Y. (2021). Fast Complex-Valued CNN for Radar Jamming Signal Recognition. Remote Sens..

[42] Shi J., Hong S., Cai C., Wang Y., Huang H., Gui G. (2020). Deep Learning-Based Automatic Modulation Recognition Method in the Presence of Phase Offset. IEEE Access.

[43] Wang T., Hou Y., Zhang H., Guo Z. (2021). Deep Learning Based Modulation Recognition With Multi-Cue Fusion. IEEE Wirel. Commun. Lett..

